# Organic amendments improve salinity-induced osmotic and oxidative stress tolerance in Okra (*Abelmoschus esculentus* (L.)Moench)

**DOI:** 10.1186/s12870-023-04527-x

**Published:** 2023-10-27

**Authors:** Alia Naseem, Sumera Iqbal, Khajista Jabeen, Aisha Umar, Khadiga Alharbi, Mohammed Antar, Katarzyna Grądecka-Jakubowska, Marek Gancarz, Iftikhar Ali

**Affiliations:** 1 Department of Botany, Lahore College for Women University, Lahore, Pakistan; 2 Institute of Botany, University of the Punjab, Lahore, Pakistan; 3 Department of Biology, College of science, Princess Nourah bint Abdulrahman University, P.O.Box 84428, Riyadh, 11671 Saudi Arabia; 4 Department of Plant Science, McGill University, Sainte-Anne-de-Bellevue, Quebec, H9X 3V9 Canada; 5 Faculty of Production and Power Engineering, University of Agriculture in Krakow, Balicka 116B, Krakow, 30-149 Poland; 6 Institute of Agrophysics, Polish Academy of Sciences, Doświadczalna 4, Lublin, 20-290 Poland; 7 Department of Genetics and Development, Columbia University Irving Medical Center, New York, NY 10032 USA; 8 School of Life Sciences & Center of Novel Biomaterials, The Chinese University of Hong Kong, Shatin, Hong Kong

**Keywords:** Okra, Organic amendments, Salinity stress, Compost, Farmyard manure, Press mud

## Abstract

**Aims:**

Salinity adversely affects okra [*Abelmoschus esculentus* (L.) Moench] plants by inducing osmotic and oxidative stresses. This study was designed to enhance salinity-induced osmotic and oxidative stress tolerance in okra plants by applying organic amendments.

**Methods:**

The effects of different organic amendments (municipal solid waste compost, farmyard manure (FYM) and press mud) on osmotic potential, water use efficiency, activities of antioxidant enzymes, total soluble sugar, total soluble proline, total soluble protein and malondialdehyde (MDA) contents of okra plants grown under saline conditions (50 mM sodium chloride) were evaluated in a pot experiment. The organic amendments were applied each at the rate of 5% and 10% per pot or in various combinations (compost + FYM, FYM + press mud and compost + press mud each at the rate of 2.5% and 5% per pot).

**Results:**

As compared to control, high total soluble sugar (60.41), total soluble proline (33.88%) and MDA (51%) contents and increased activities of antioxidant enzymes [superoxide dismutase (83.54%), catalase (78.61%), peroxidase (53.57%] in salinity-stressed okra plants, were indicative of oxidative stress. Salinity significantly reduced the osmotic potential (41.78%) and water use efficiency (4.75%) of okra plants compared to control. Under saline conditions, 5% (farmyard manure + press mud) was the most effective treatment, which significantly improved osmotic potential (27.05%), total soluble sugar (4.20%), total soluble protein (73.62%) and total soluble proline (23.20%) contents and superoxide dismutase activity (32.41%), compared to saline soil. Application of 2.5% (FYM + press mud), 5% press mud, and 10% compost significantly reduced MDA content (27%) and improved activities of catalase (38.64%) and peroxidase (48.29%), respectively, compared to saline soil, thus facilitated to alleviate oxidative stress in okra plants.

**Conclusions:**

Using organic amendments (municipal solid waste compost, farmyard manure and press mud) was a cost-effective approach to improve salinity-induced osmotic and oxidative stress tolerance in okra plants.

## Introduction

Salinity is a devastating abiotic stress and a global issue for crop production and agriculture sustainability [1]. Salinity stress interrupts plants’ metabolic activities by inducing osmotic stress pressure due to increased solute concentration and specific ion effect or ion toxicity instigating the secondary stress, i.e., oxidative stress in plants [2, 3]. Salinity-induced osmotic and oxidative stresses reduce the photosynthetic efficiency and cause damage to plant proteins and membranes [4, 5].

Salinity stress disturbs enzymatic activities and nutrient homeostasis, causing a substantial reduction in plant growth and production [6]. Under saline conditions, increased lipid peroxidation [7] enhances the membrane permeability with subsequent outflow of the ions from cells [8]. This leads to osmotic and ionic stresses as well as the over-production of reactive oxygen species (ROS), resulting in oxidative stress [9, 10]. The ability of plants to detoxify ROS provides protection against oxidative stress [11].

Plants cope with the adverse effects of salinity by switching on adaptive mechanisms such as increasing osmolyte accumulation and activities of antioxidant enzymes, which decrease the sodium ion (Na^+^) absorption [12]. Plant salinity tolerance is correlated with antioxidant system stimulation and oxidative impairment attenuation [13]. Under salinity stress, enhanced activities of antioxidants in plants indicate the remediation of oxidative stress through ROS quenching [14, 15]. The primary antioxidant enzymes, e.g. superoxide dismutase and peroxidase, are produced in plants under saline conditions [16]. The osmolytes function as cytoplasmic osmoregulators and mitigate oxidative stress under salty conditions [17]. Proline accumulation in plants is ascribed to increased salinity tolerance [1]. Accumulation of soluble sugars in response to salinity stress regulates the structural growth of plants and contributes to alleviating the adverse impacts of salinity [18].

Using recycled organic waste products for soil fertility improvment and crop productivity has been used as a conventional agriculture approach for years [19]. Compared to high-priced inorganic fertilizers, applying organic amendments, such as press mud, farmyard manure, and green manure, is considered a suitable and cost-effective method for the reclamation of saline-sodic soils [20, 21]. The application of organic amendments alleviates salinity stress through the induction of various mechanisms such as diminution of oxidative stress, maintaining ionic equilibrium and induction of antioxidant enzyme system.

Okra (*Abelmoschus esculentus*) is a highly nutritious vegetable with reduced optimum yield per hectare due to salinity-induced osmotic and oxidative stresses. The detrimental impacts of salinity on physiological attributes and metabolic and enzymatic activities of okra plants have been documented previously [22, 23]. Several studies have been conducted to alleviate salinity stress in okra by application of different organic amendments. However, there is a need to investigate the ameliorative influence of locally available, low cost and eco-friendly organic amendments and their combinations on osmotic and oxidative stresses induced by salinity in okra. The combinations might have different impacts compared to an individual application.

Thus, the primary aim of this research work was to improve the salinity tolerance of okra by applying organic amendment(s) or their combination by alleviating salinity-induced osmotic and oxidative stresses. Due to the local availability of municipal solid waste compost, FYM and press mud, this research might provide a feasible and economical approach for small-scale farmers to increase okra productivity under saline conditions and promote organic agriculture.

## Materials and methods

### Plant material and seed sterilization

Okra (*Abelmoschus esculentus* (L.) Moench var. Swat Green was used as plant material in this experiment. Seeds of okra plants were acquired from the National Agricultural Research Council, Islamabad (Pakistan) and kept in air-tight bags. Before sowing, healthy okra seeds were sorted out and surface sterilized to prevent microbial infection. For surface sterilization, seeds were soaked in 0.2% sodium hypochlorite solution for about 20 min, with subsequent rinsing with distilled water. Afterwards, the seeds were dried on aseptic blotting paper sheets.

### Experimental design

In total, 28 treatments, with 3 replicates, were distributed in a completely randomized design (CRD). The soil used in this experiment was air-dried and passed through a 0.5 mm soil siever. Each pot was filled with 7 kg of soil prior to sowing 10 okra seeds. In half of the pots, salinity stress was applied by adding sodium chloride (Analytical Grade, Merk) at the rate of 50 mM per pot. The organic amendments of municipal solid waste compost, farmyard manure (FYM) and press mud were applied each at the rate of 5% and 10% per pot with various combinations (compost + FYM, FYM + press mud and compost + press mud each @ 2.5% and 5% per pot). The application of organic amendments was carried out 1 month before sowing seeds. The recommended dose of NPK fertilizer (90-45-45) was added in the form of urea, diammonium phosphate and potassium sulphate in 2 treatments (T2 (non-saline) and T16 (saline). The control plants were left without any treatment. On alternate days, plant irrigation was done using tap water. The treatments used in the experiments are given in Table 1. For determination of MDA content and activities of antioxidant enzymes in okra leaves, treatments of control (T1), saline (T15), NPK (T16), 10% compost (T18), 5% press mud (T21), 2.5% (FYM + press mud) (T25) and 5% (FYM + press mud) (T26) were selected. After 2 weeks of seed germination, thinning was performed to keep only 5 plants in each pot. For plant sampling, 3 plants were collected randomly from each treatment, cleaned carefully, and preserved in labelled paper bags.

**Table 1:** List of treatments applied in the experiment

Sr No.	Non-saline soil	Sr No.	Saline soil
T1	Control	T15	Saline soil
T2	NPK	T16	NPK
T3	5% Compost	T17	5% Compost
T4	10% Compost	T18	10% Compost
T5	5% FYM	T19	5% FYM
T6	10% FYM	T20	10% FYM
T7	5% Press mud	T21	5% Press mud
T8	10% Press mud	T22	10% Press mud
T9	2.5% Compost + 2.5% FYM	T23	2.5% Compost + 2.5% FYM
T10	5% Compost + 5% FYM	T24	5% Compost + 5% FYM
T11	2.5% FYM + 2.5% Press mud	T25	2.5% FYM + 2.5% Press mud
T12	5% FYM + 5% Press mud	T26	5% FYM + 5% Press mud
T13	2.5% Compost + 2.5% Press mud	T27	2.5% Compost + 2.5% Press mud
T14	5% compost + 5% Press mud	T28	5% compost + 5% Press mud

### Soil analysis

The soil physical and chemical properties (Tables 2 and 3) were determined using the standard methods. The soil samples taken from each treatment were heated in an electric oven (Hinotech, GX30B WHL-25 A) at 80 °C for 3 days before storing it for further analysis. The properties of organic amendments, i.e. compost, FYM and press mud, were also determined and mentioned in Table 4.

**Table 2:** Soil physical and chemical characteristics

Clay 38.2%	Silt 36. 5%	Sand 25.3%	Soil textural class	Clay loam							
**Non-saline soil**	**pH**	**E.C.** **(dS/m)**	**TSS** **(ppm)**	**OM** **(%)**	**BD** **(g/cm** ^**3**^ **)**	**Saline soil**	**pH**	**E.C.** **(dS/m)**	**TSS** **(ppm)**	**OM** **(%)**	**BD** **(g/cm** ^**3**^ **)**
Control	7.77	0.76	484.27	0.53	1.31	Saline soil	7.75	4.85	3106.13	0.48	1.30
NPK	7.79	0.78	501.33	0.55	1.3	NPK	7.77	4.72	3018.67	0.51	1.29
5% Comp.	7.45	0.69	441.60	0.62	1.29	5% Comp	7.62	2.53	1619.20	0.59	1.27
10% Comp.	7.69	0.63	401.07	0.63	1.29	10% Comp.	7.62	2.36	1508.27	0.60	1.27
5% FYM	7.47	0.68	437.33	0.60	1.27	5% FYM	7.5	2.60	1666.13	0.56	1.26
10% FYM	7.62	0.65	413.87	0.61	1.28	10% FYM	7.63	2.42	1546.67	0.57	1.27
5% PM	7.67	0.71	454.40	0.59	1.27	5% PM	7.61	2.61	1672.53	0.57	1.26
10% PM	7.69	0.66	422.40	0.61	1.27	10% PM	7.65	2.57	1646.93	0.59	1.27
2.5% (C + F)	7.64	0.62	394.67	0.64	1.28	2.5% (C + F)	7.67	2.4	1533.87	0.61	1.27
5% (C + F)	7.63	0.60	381.87	0.65	1.28	5% (C + F)	7.65	2.32	1484.80	0.63	1.27
2.5% (F + P)	7.64	0.63	405.33	0.63	1.28	2.5% (F + P)	7.59	2.34	1495.47	0.61	1.26
5% (F + P)	7.60	0.61	390.40	0.64	1.29	5% (F + P)	7.63	2.29	1465.6	0.62	1.26
2.5% (C + P)	7.63	0.64	411.73	0.63	1.29	2.5% (C + P)	7.60	2.43	1555.20	0.59	1.28
5% (C + P)	7.61	0.62	398.93	0.63	1.29	5% (C + P)	7.62	2.41	1542.40	0.61	1.28

*EC* electrical conductivity, *TSS* total soluble salts, *OM* organic matter content, *BD.* bulk density, *Comp., C* compost, *FYM* F farmyard manure, *PM P* press mud, *dS/m* desi Siemens per metre, *ppm* parts per million, *g/cm*^*3*^gram per cubic centimetre

**Table 3:** Soil macronutrient and micronutrient analysis

	Control	Saline	S + NPK	S + 10% Comp.	S + 5% PM	S + 2.5% FYM + 2.5% PM	S + 5% FYM + 5% PM
**N (%)**	0.145 d	0.135	0.148 cd	0.152 bc	0.148 cd	0.160 a	0.155 ab
**P (%)**	0.010 c	0.002 d	0.015 b	0.02 a	0.015 b	0.022 a	0.018 ab
**OC (%)**	0.318 d	0.270 e	0.314 d	0.348 b	0.330 c	0.353 ab	0.359 a
**C:N**	2.19 bc	2.00 d	2.12 c	2.29 ab	2.23 abc	2.21 bc	2.32 a
**K** ^**+**^ **(meq/L)**	15.24 d	7.48 e	22.70 c	28.72 b	24.45 c	32.46 a	27.18 b
**Na** ^**+**^ **(meq/L)**	13.2 e	29.6 a	21.34 b	18.45 c	20.73 b	16.42 d	19.78 bc
**K** ^**+**^ **/Na** ^**+**^	1.16 d	0.25 e	1.07 d	1.56 b	1.18 d	1.98 a	1.38 c
**Ca** ^**2+**^ **(meq/L)**	15.9 c	11.8 d	16.35 c	22.37 b	21.56 b	24.18 a	24.63 a
**Mg** ^**2+**^ **(meq/L)**	9.4 e	5.4 f	10.82 d	16.05 a	11.08 d	12.73 c	14.55 b
**SAR**	3.71 e	10.01 a	5.79 b	4.22 de	5.13 c	3.82 e	4.47 d
**ESP (%)**	4.03 f	11.89 a	6.78 b	4.72 de	5.93 c	4.19 of	5.06 d
**Cu (ppm)**	32.54 d	25.31 e	36.20 cd	62.48 a	38.61 c	52.75 b	49.73 b
**Zn (ppm)**	112.73 e	104.64 f	178.22 d	264.58 c	319.32 b	365.14 a	318.29 b
**Fe (ppm)**	14.85 e	8.57 f	23.16 d	32.59 bc	29.10 c	41.32 a	36.21 b
**Mn (ppm)**	15.4 f	11.2 g	18.90 e	25.71 c	22.46 d	28.74 b	32.58 a

*N*  Nitrogen, *P* Phosphorus, *OC* organic carbon, *K* Potassium, *Na* Sodium, *Ca*^*2+*^ Calcium, *Mg*^*2+*^ Magnesium, *SAR* Sodium Absorption Ratio, *ESP* Exchangeable Sodium Percentage, *Cu* Copper, *Zn* zinc, *Fe* Iron, *Mn* Manganese, *ppm*  parts per million, *meq/L* milliequivalent per litre. The values with the same letters are not significantly different at *p* < 0.05

**Table 4:** Analysis of organic amendments

	Compost	FYM	Press mud
pH	6.5	7.6	7.8
EC (dS/m)	8.4	3.5	2.3
Organic matter (%)	42	28	35
Organic carbon (%)	24.36	16.24	20.30
Total Nitrogen (%)	1.36	1.5	2
Phosphorus (%)	0.278	0.2	1.34
Potassium (%)	0.52	0.57	1.5
C/N	17.91	10.82	10.15

*EC* Electrical conductivity, *C/N* Carbon/Nitrogen ratio, *dS/m* desiSiemens per metre

### Determination of osmotic potential

The osmotic potential of okra leaves was determined (30 days after germination of seeds) following the Capell and Doerffling [24] method, using a vapour pressure osmometer (Wescor model VAPRO 5520). Okra leaves were enclosed in plastic syringes and kept in a freezer for 3 or 4 days. Cell sap was squeezed out of leaves in a container by pressing syringes, and about 50 µL of sap was collected with a micropipette. The osmolality values (mmol Kg^‒1^) of cell sap samples were recorded with the help of a vapour pressure osmometer. The osmotic potential of leaves (MPa) was estimated by using the following Eq.

1 $$Osmotic\;potential=\;Osmolality\;x\;0.831\;\times\;10^{-5}\;x\;T\;K$$

Where,

T = thermodynamic temperature (T = 273 + t°C) expressed in K

### Determination of gas exchange parameters

The gas exchange parameters (photosynthesis rate and transpiration rate) of okra leaves were determined 30 days after seed germination using portable IRGA (Infra-red gas analyzer, LI-6400 XT LI-COR Inc., Lincoln, NE, USA). The intact leaves were placed carefully in the leaf chamber of IRGA. Gas exchange parameters were determined in the morning time (9.00 to 11.00 AM) with the following requirements: PAR (photosynthetically active radiation), 950 µmol m^−2^ s^−1^, the temperature of leaf chamber, 32–38 °C; atmospheric CO_2_ concentration, 450 µmol mol^−1^, and atmospheric pressure, 980 mbars. The following equation was used to calculate water use efficiency.

2 $$WUE\;=\;\frac{P_{net}}E$$

Where,

WUE = Water use efficiency

P_net_ = net photosynthetic rate

E = transpiration rate

### Determination of total soluble sugar content

The total soluble sugar content in okra leaves was determined (30 days after germination of seeds) using the method of Dubois et al. [25]. 0.5 g leaves were finely crushed using a pestle and mortar. Then, 1 mL of distilled water was mixed with crushed leaves and filtered. The collected filtrate (0.1 mL) was mixed with 1 mL of 5% phenol solution in a test tube. After 1 h of incubation at room temperature, 5 mL of concentrated Sulphuric acid was added to it. Each sample was separately transferred to a quartz cuvette to check the absorbance at 420 nm using a UV-vis spectrophotometer (Shimadzu UV-2600 BMS). The total soluble sugar content of leaves was estimated from a standard curve prepared for glucose solutions of different known concentrations (5, 10, 15, 20 and 25 mg mL^−1^).

### Estimation of total soluble proline content

The total soluble proline content in the okra leaves was determined 30 days after seed germination following the method of Bates et al. [26]. The 0.1 g of 2nd or 3rd leaves was homogenized thoroughly with 4 mL sulphosalicyclic acid (3%) using a pestle and mortar and incubated for 24 h at 5 °C. Afterwards, samples were centrifuged at 10,000 × g for 5 min, and supernatants were collected. Then, 2 mL supernatant was mixed with 4 mL of acidic ninhydrin reagent in a test tube, followed by vigorous shaking. The mixture was heated in a boiling water bath (100 °C) for 1 h. After cooling, 4 mL toluene was poured into the mixture and shaken well. The toluene layer was transferred to a cuvette, and absorbance was measured at 520 nm using a UV-vis spectrophotometer. The total soluble proline content in okra leaves was estimated from the standard curve for proline solutions of known concentrations (10, 20, 30, 40 and 50 µM).

### Determination of total soluble protein content

The total soluble protein content in okra leaves was determined 30 days after seed germination following Lowry et al.‘s method [27]. 0.4 g sodium hydroxide, 2 g sodium carbonate and 1 g sodium potassium tartrate were dissolved into 100 mL distilled water to prepare reagent (A) 0.5 g copper sulphate was mixed with 100 mL distilled water to prepare reagent (B) 50 mL reagent A and 1 mL reagent B were mixed to prepare reagent (C) To prepare reagent D, 3 mL Folin phenol reagent and 3 mL distilled water (1:1) were mixed.

The finely crushed 0.2 g of okra leaves were mixed with 1 mL sodium phosphate buffer (pH 7.5) solution. The mixture was centrifuged (10,000 × g) and filtered. Approximately 0.1 mL filtrate was poured into a test tube, and volume was raised up to 1 mL by adding 0.9 mL distilled water. After that, 1 mL reagent C was added to the mixture and stirred for 10 min. Then 0.1 mL reagent D was dissolved into the solution and incubated for 30 min. The reaction mixture from each sample was poured into a quartz cuvette, and absorbance was recorded by using a UV-vis spectrophotometer. The total soluble protein content in okra leaves was estimated from a standard curve prepared with known concentrations (2, 4, 6, 8, 10 mg mL^‒1^) of bovine serum albumin solutions. For selected treatments antioxdant analysis was performed.

### Estimation of Malondialdehyde (MDA) content

Thiobarbituric acid reactive substances (TBARS) are formed due to lipid peroxidation. The evaluation of TBARS concentration is indicative of the level of lipid peroxidation [28]. The crushed okra leaves (0.1 g) were homogenized with 0.2 mL trichloroacetic acid (0.1%), centrifuged at 10,000 × g (15 min) and filtered. In a test tube, 4 mL thiobarbituric acid (0.5%) and 1 mL trichloroacetic acid (20%) were mixed with 1 mL of the above filtrate. The test tubes were heated in a water bath (95 °C) for half an hour. After that, the mixture was cooled down in an ice bath, centrifuged at 10,000 × g (10 min) and filtered. The absorbance of the filtrate was noted at different wavelengths of 440, 532, and 600 nm using a double-beam UV-vis spectrophotometer. Melondialdehyde equivalents were calculated using the Du and Bramlage formula [29].

3 $$\left[\left(A532-A600\right)\;-\left\{\left(A440-A600\right)\;\frac{\left(MA\;of\;sucrose\;at\;532\;nm\right)}{MA\;of\;sucrose\;at\;440\;nm}\right\}\right]\;/\;157000\rbrack\;\;\times\;10^6$$

### Determination of antioxidant enzyme activities

#### Preparation of enzyme extract

To prepare enzyme extract, 0.5 g okra leaves were homogenized with 5 mL of 50 mM potassium phosphate buffer solution (pH: 7.00) in an ice bath. The mixture was filtered after centrifugation (10,000 × g) at 4(C for about 20 min. The filtrate was used as an enzyme extract to estimate antioxidant enzyme activities.

### Determination of superoxide dismutase activity

The activity of SOD (superoxide dismutase) is a measure of enzyme capability to suppress the photochemical reduction of nitroblue tetrazolium. Beauchamp and Fridovich [30] method was used to estimate SOD activity in okra leaves. For preparation of 2 mL reaction mixture, 0.5 mL phosphate buffer (50 mM, pH: 7.8), 0.2 mL methionine (13 mM), 0.1 mL nitroblue tetrazolium (0.075 mM), 0.1 mM EDTA, 0.2 mL triton X, and 0.1 mL riboflavin (0.002 mM) mixed with 0.1 mL of enzyme extract. The test samples were illuminated by ultraviolet light for 15 min while the control sample remained non-irradiated. The absorbance of sample solutions was noted at 560 nm by using a spectrophotometer. One unit of SOD is the amount of enzyme capable of repressing 50% absorbance relative to control.

4 $$IU=\;\frac{absorbance}{50}\;x\;10$$

Where,

IU = International unit of enzyme activity

5 $$SOD\;activity\;=\;\frac{IU}{mg\;of\;protein}$$

### Determination of catalase activity

Teranishi et al. [31] method was followed to determine catalase (CAT) activity in okra leaves. For preparing the reaction mixture (3 mL), 2.6 mL phosphate buffer (50 mM, pH 7.2) and 0.2 mL H_2_O_2_ (15 mM) were mixed with 0.2 mL of enzyme extract. The reaction was stopped after 5 min when 3 mL titanium reagent was added to the mixture, which reacted with the available H_2_O_2_ to form a yellow complex. The reaction mixture was centrifuged (10,000 × g), filtered, and absorbance was recorded at 410 nm using a a spectrophotometer. Patty and Bonet Maury’s [32] method, as modified by Teranishi et al. [31], was followed to prepare titanium reagents. 1 g titanium oxide and 10 g potassium sulphate were mixed with 10 mL concentrated Sulphuric acid. The mixture was heated on a heating mantle for 2 h. The digested mixture was cooled down, and 1.5 L of distilled water was added to dilute it.

6 $$CAT\;activity\;=\;\frac{\triangle\;410}{mg\;of\;protein}$$

∆ 410 = The variations in absorbance readily recorded at 410 nm after the reaction between enzyme extract and oxidants.

### Determination of peroxidase activity

Peroxidase (POD) activity in okra leaves was estimated according to the method of Vetter et al. [33] as modified by Gorin and Heidema [34]. For preparing the reaction mixture, 1.8 mL phosphate buffer (100 mM, pH: 7), 0.3 mL H_2_O_2_ (3mM) and 0.1 mL aqueous solution of (1%) ρ- phenylenediamine (w/v) were mixed with 0.2 mL of enzyme extract. The absorbance alterations of individual samples taken in the cuvette were traced for 3 min at 485 nm. One unit of POD was estimated by calculating mg of protein using a standard curve.

7 $$POD\;activity\;=\;\frac{\triangle\;485}{mg\;of\;protein}$$

### Statistical analysis

The data collected from each treatment was expressed as mean ± SE. All the data was statistically analyzed through analytical software [35]. Statistics (ver. 8.1, 2005) and means were compared by Least Significant Difference. The statistical analysis (variance, simple correlations and principal component analysis) was done at the significance level α = 0.05 using TIBCO Statistica software (version 12.0, StatSoft Inc., Palo Alto, CA, USA). The principal component analysis was applied to find the associations between cases and compounds. The PCA data matrix for the statistical analysis of results had 9 columns (names of the compounds) and 21 rows (type of case). The input matrix was scaled automatically. The optimal number of principal components obtained in the analysis was determined based on the Cattel criterion.

## Results

**Fig. 1:**
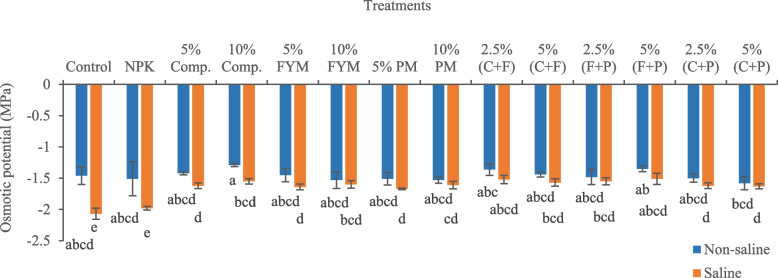
Impact of treatments on osmotic potential of okra plants grown in saline and non-saline soil. (Comp., C = compost, FYM, F = farmyard manure, PM, P = press mud, MPa = megapascals). The means sharing similar letters are not significantly different

**Fig. 2:**
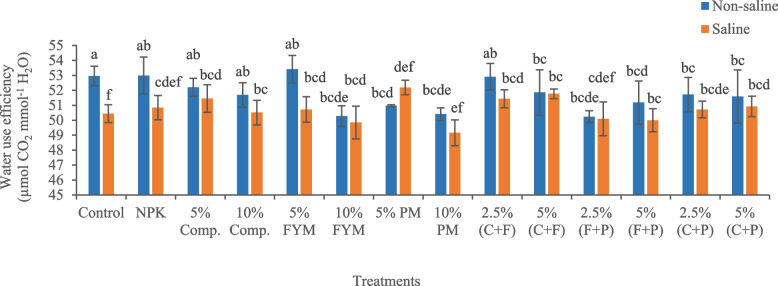
Impact of treatments on water use efficiency of okra plants grown in saline and non-saline soil. (Comp., C = compost, FYM, F = farmyard manure, PM, P = press mud, CO_2_ = carbon dioxide, H_2_O = water, µmol = micromole, mmol = millimole). The means sharing similar letters are not significantly different

The imposition of salinity stress significantly (*p* < 0.05) reduced osmotic potential (41.78%) in okra plants relative to control (Fig. 1). In saline conditions, the addition of all organic amendments significantly improved the osmotic potential of plants. The amender 5% (FYM + press mud) was the most effective treatment, which significantly (*p* < 0.05) improved the osmotic potential (27.05%) of plant leaves compared to saline soil. Salinity significantly (*p* < 0.05) reduced water use efficiency (4.75%) of okra plants, relative to control (Fig. 2). In saline soil, the maximum increase in water use efficiency (3.46%) of okra plants was with application of 5% press mud.

**Fig. 3:**
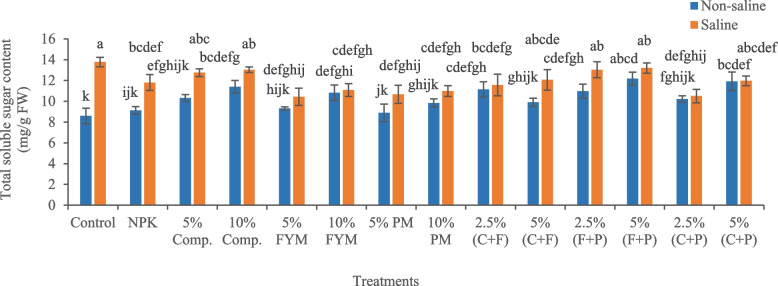
Impact of treatments on leaf total soluble sugar content of okra plants grown in saline and non-saline soil. (Comp., C = compost, FYM, F = farmyard manure, PM, P = press mud, mg/g milligram per gram, FW = fresh weight). The means sharing similar letters are not significantly different

**Fig. 4:**
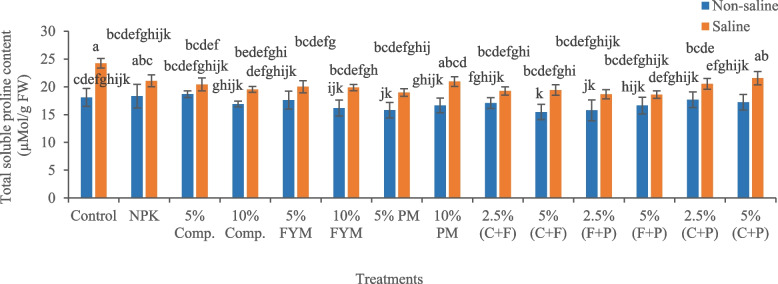
Impact of treatments on leaf total soluble proline content of okra plants grown in saline and non-saline soil. (Comp., C = compost, FYM, F = farmyard manure, PM, P = press mud, µMol/g = micromole per gram, FW = fresh weight). The means sharing similar letters are not significantly different

Salinity stress significantly (*p* < 0.05) increased total soluble sugar content (60.41%) in okra plants compared to control (Fig. 3). Under saline conditions, amender 5% (FYM + press mud) effectively decreased total soluble sugar content (4.20%) in plants, compared to salty soil. The highest total soluble proline content in salinity-stressed plants indicated oxidative stress. Salinity stress significantly (*p* < 0.05) increased total soluble proline content (33.88%) in okra plants, compared to control (Fig. 4). Under saline conditions, amender 5% (FYM + press mud) was the most effective treatment, which significantly (*p* < 0.05) decreased total soluble proline content (23.20%) in okra compared to saline soil, thus facilitated to alleviate salinity stress in plants.

**Fig. 5:**
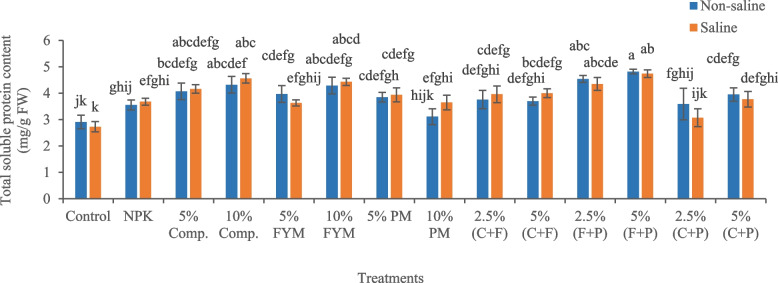
Impact of treatments on leaf total soluble protein content of okra plants grown in saline and non-saline soil. (Comp., C = compost, FYM, F = farmyard manure, PM, P = press mud, mg/g = milligram per gram, FW = fresh weight). The means sharing similar letters are not significantly different

**Fig. 6:**
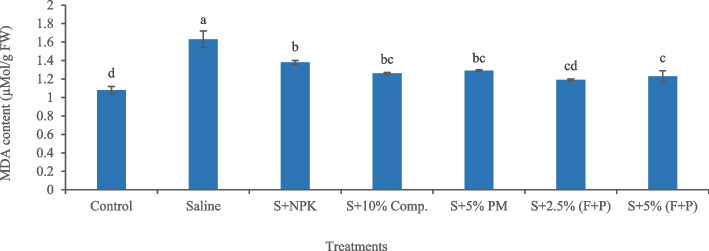
Impact of treatments on leaf MDA content of okra plants grown in saline and non-saline soil. (Comp. = compost, F = farmyard manure, PM, P = press mud, MDA = melondialdehyde, S = saline, µMol/g = micromole per gram, FW = fresh weight). The means sharing similar letters are not significantly different

The imposition of salinity stress decreased total soluble protein content (6.18%) in okra plants, compared with control (Fig. 5). Under saline conditions, 5% (FYM + press mud) was an effective treatment, which significantly (*p* < 0.05) increased total soluble protein content (73.62%) in plants, compared to saline soil. Salinity significantly (*p* < 0.05) increased MDA content (51%) in okra plants, compared with control, indicating oxidative stress (Fig. 6). Under saline conditions, 2.5% (FYM + press mud) was the most effective treatment, which significantly (*p* < 0.05) reduced MDA content (27%) in plants compared to saline soil, thus contributed to alleviating oxidative stress.

**Fig. 7:**
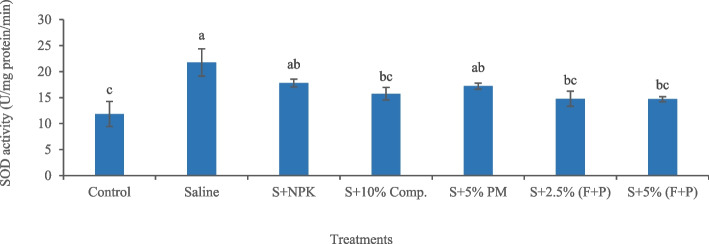
Impact of treatments on SOD activity in leaves of okra plants grown in saline and non-saline soil. (Comp. = compost, F = farmyard manure, PM, P = press mud, SOD = superoxide dismutase, S = saline, U = enzyme activity, mg/min = milligram per minute). The means sharing similar letters are not significantly different

**Fig. 8:**
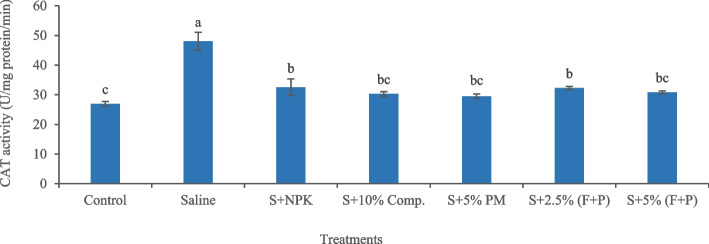
Impact of treatments on CAT activity in leaves of okra plants grown in saline and non-saline soil. (Comp. = compost, F = farmyard manure, PM, P = press mud, CAT = catalase, S = saline, U = enzyme activity, mg/min = milligram per minute). The means sharing similar letters are not significantly different

**Fig. 9:**
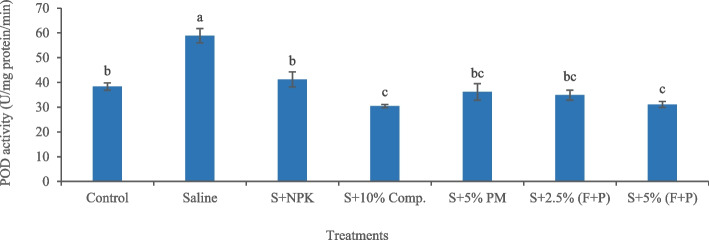
Impact of treatments on POD activity in leaves of okra plants grown in saline and non-saline soil. (Comp. = compost, F = farmyard manure, PM, P = press mud, POD = peroxidase, S = saline, U = enzyme activity, mg/min = milligram per minute). The means sharing similar letters are not significantly different

Salinity-induced increase in antioxidant enzyme activities indicated oxidative stress in okra plants. Salinity significantly (*p* < 0.05) increased SOD activity (83.54%) in plants compared with control (Fig. 7). Under saline conditions, 5% (FYM + press mud) was an effective treatment in reducing SOD activity (32.41%) in plants compared to salty soil. Similarly, salinity significantly (*p* < 0.05) increased CAT activity (78.61%) in okra plants compared with control (Fig. 8). Under saline conditions, 5% press mud was the most effective treatment, which significantly (*p* < 0.05) decreased CAT activity (38.64%) in plants compared to saline soil. Salinity significantly (*p* < 0.05) increased POD activity (53.57%) in plants compared with control (Fig. 9). Under salty conditions, 10% compost was the most effective treatment, which significantly (*p* < 0.05) reduced POD activity (48.29%) in plants compared to saline soil.

**Fig. 10:**
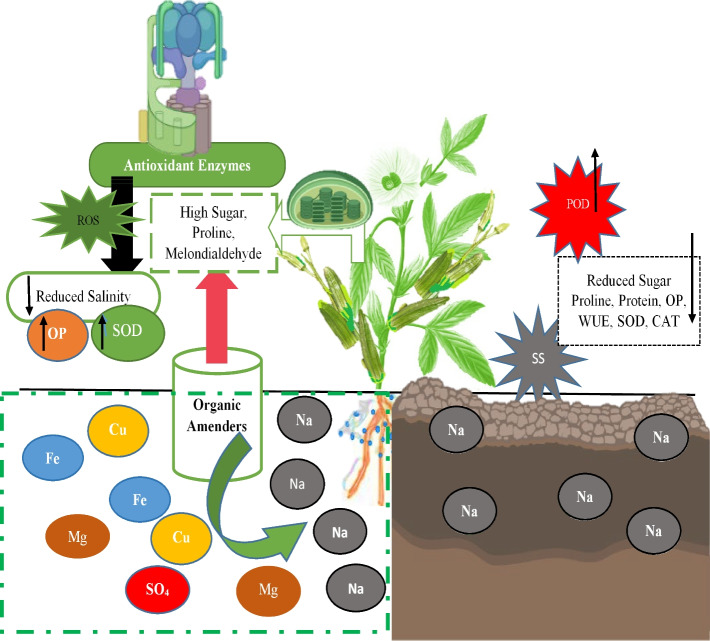
The mechanism of salinity reduction by organic amenders facilitate the higher sugar, proline and melondialdehyde contents. This leads to production of antioxidant enzymes and ROS, which ultimately reduced the salinity stress by increasing the oxidative potential, SOD, and water use efficiency. The saline soil reduced all these contents, but highly favors the POD activity in okra leaves

**Fig. 11:**
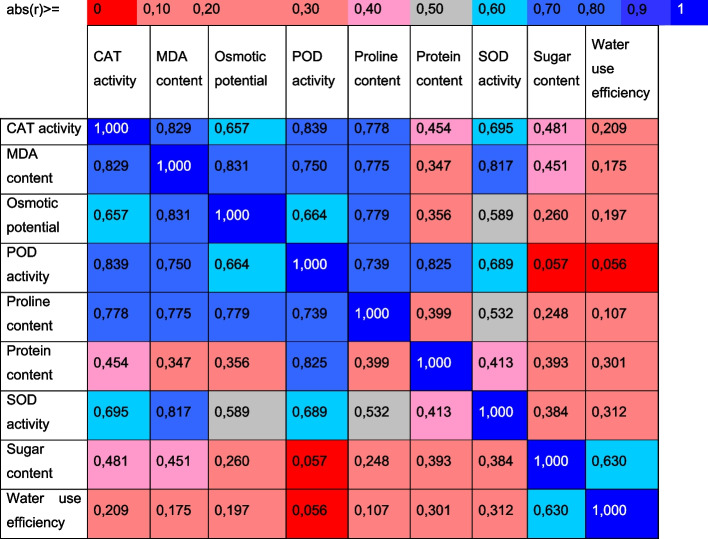
Correlation matrix for the tested parameters

**Fig. 12:**
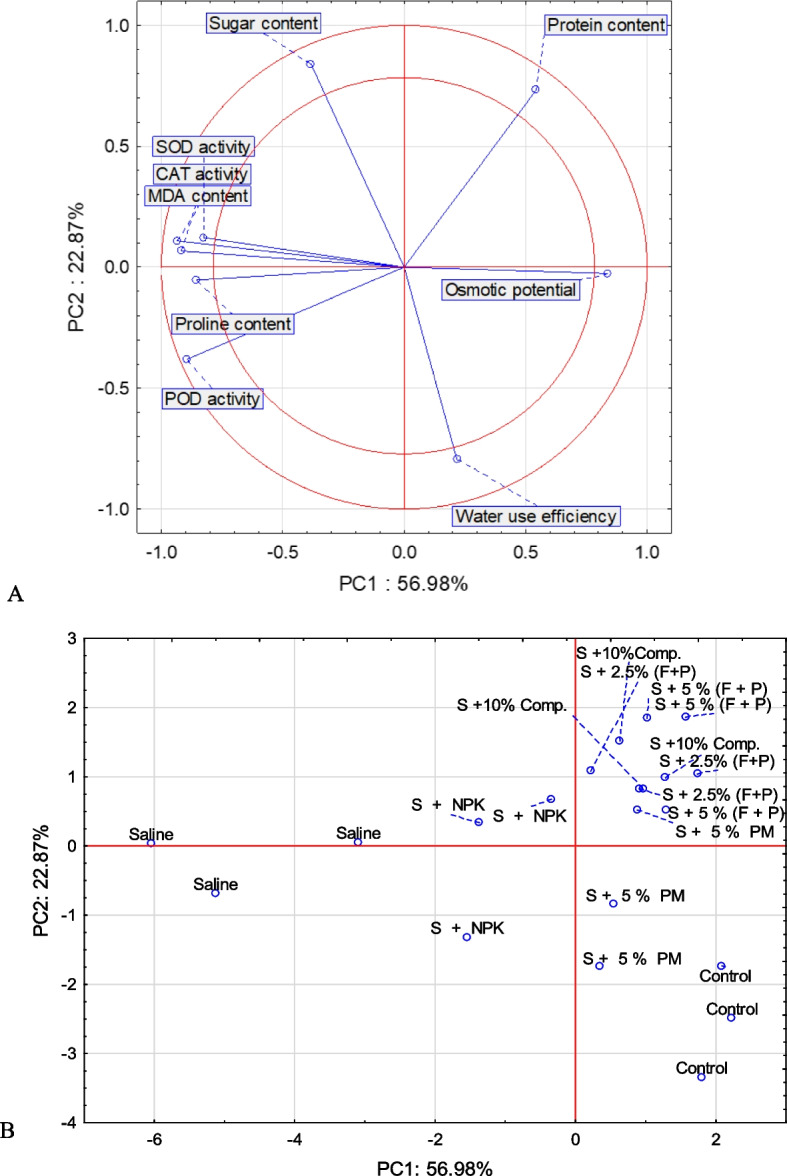
Projection of variables: compounds on the PC1 and PC2 loadings plot—(**A**); projection of cases on the PC1 and PC2 scores plot—(**B**)

The effects of organic amenders are shown in mechanism form, because salinity badly reduced the biochemical contents of okra in this study. The production rate was increased by a special type of amender used in this study (Fig. 10). Figure 11 shows the correlation matrix for the tested parameters. The correlation matrix’s determinant defines the explanatory variables’ collinearity (correlation) in which the closer to 0, the lower the degree of mutual correlation of explanatory variables. The closer to 1, the stronger the correlation. Employing the principal components analysis (PCA) allowed for obtaining nine new variables, components explaining the system’s variability (Fig. 12A and B). Figure 12A and B show the variables’ projection on planes PC1 (56.98%) and PC2 (22.87%), which describe the dependencies at 79.90%. A strong positive correlation was found between SOD activity, CAT activity, POD activity, MDA content and proline content. The correlation between these parameters and osmotic potential was strong and negative. A negative but weak correlation occurs between SOD activity, CAT activity, POD activity, MDA content, proline content and protein content. The correlation between sugar content and water use efficiency was stronger and negative. In turn, there was no correlation between SOD activity, CAT activity, POD activity, MDA content, proline content, osmotic potential, sugar content and water use efficiency. All compounds within the two-circle region strongly influence the variability of the system (Fig. 12A). Figure 12B shows cases. Positive PC1 values and positive PC2 values described the cases: S + 10% comp., S + 2.5% (F + P) and S + 5% (F + P), but negative PC1 values described the saline and S + NPK cases. In turn, the positive PC1 values and negative second principal component (PC2) explained the case of control and S + 5% PM (Fig. 12B).

In general, the first principal component (PC1) explain relationship between saline, S + NPK, and the cases: S + 10% comp., S + 2.5% (F + P), S + 5% (F + P), S + 5% PM and control. In turn, the second main component (PC2) describe relationship between control and S + 5% PM, and cases: S + 10% comp., S + 2.5% (F + P), S + 5% (F + P).

The MDA content and the activity of SOD, CAT and POD describe the saline; the water use efficiency describes control, and the protein content describes S + 10% comp., S + 2.5% (F + P) and S + 5% (F + P).

## Discussion

The accretion of soluble salts in the root zone under osmotic stress lowers the soil water potential, thus impeding water absorption and nutrient uptake by plant roots [3]. Ionic stress drives a massive inflow of sodium ions, causing ample outflow of potassium ions [36, 37]. Salinity stress induces alterations in atmospheric vapour pressure and leaf turgor pressure due to the limited opening of stomata [38]. Hence, the retention of mesophyll cells and restricted stomatal opening in response to stress conditions lead to declined net photosynthetic rate [39]. Water use efficiency and leaf water potential are governed by the combined effects of net photosynthetic rate and stomatal conductance [40]. These changes adversely affect plant biochemical and physiological processes, resulting in impaired water relations [41], oxidative stress [42], depressed plant growth and declined productivity [43].

Salinity-induced secondary stress in plants is called oxidative stress, which destroys cell performance [44]. Under salinity stress, deficiency of potassium ions lowers photosynthetic rates, instigating oxidative stress, which is the major cause of reduced plant growth and productivity [45, 46]. Higher concentration of sodium ions functions as signalling molecules in transduction channels and contributes to enhanced ROS accumulation [47], which causes membrane injury and electrolyte leakage [1]. Membrane injury is the foremost impact of salinity stress indicated by the estimation of malondialdehyde (MDA) contents [47]. The results of this study showing increased antioxidant enzyme activities in response to salinity are similar to those of previous studies [3, 48]. The increased MDA content and higher antioxidant enzyme (SOD, CAT and POD) activities have been reported in lettuce plants under salinity stress [49].

Under saline conditions, incorporation of organic amendments has been found to be effective in alleviating the negative influence of oxidative stress by reducing MDA content [50] in accordance with present research. Similar to our study, the addition of organic fertilizer (vermicompost) is reported to improve antioxidant enzyme (SOD, CAT, POD) activities and reduce MDA content in tomato and maize plants grown under salinity constraints [51, 52]. The lowered electrolyte leakage by adding press mud is ascribed to reduced MDA contents due to improved activities of antioxidant enzymes and increased proline and soluble sugar [53].

Salinity-induced proline production in the cytoplasm is a vital process to manage osmotic pressure caused by cellular water deficit under salinity stress [54]. The results of this study showing higher proline content under salinity stress are similar to those in mung bean [55], wheat [56], rice [57], faba bean [42], almonds [58] and milk thistle [59]. This increases proline synthesis is considered to be by the activation of pyrroline-5-carboxylate reductase (proline synthesizing enzyme) in response to salinity stress [53].

Under abiotic stress conditions, soluble sugars are produced in plants to maintain turgor and alleviate salinity stress by acting as a carbon reservoir [60]. In addition, sugars also regulate osmotic homeostasis, shield membranes and proteins and detoxify ROS [61, 62]. Similar to the results of this study, high soluble sugar content has been reported in okra and faba beans, respectively, under salinity stress [42, 63].

The protein concentration in leaves is considered an important salinity stress marker [64]. Protein accumulation confers plant salt tolerance by controlling metabolic functions and antioxidant enzyme activities [65]. In this study, results showing the minimum protein content in okra align with previous studies [55, 56].

In okra plants, high total soluble sugar, total soluble proline and MDA contents and increased activities of antioxidant enzymes compared to control indicated the oxidative stress induced by salinity. Salinity significantly reduced okra plants’ osmotic potential and water use efficiency compared to control. Under saline conditions, 5% (FYM + press mud) was the most effective treatment, significantly improving osmotic potential, total soluble sugar, total soluble protein, total soluble proline contents and SOD activity, thus contributing to alleviating oxidative stress in okra plants. Application of organic amenders, 2.5% (FYM + press mud), 5% press mud, and 10% compost significantly reduced MDA content and improved activities of CAT and POD, respectively. The combination of these amendments seems to be more effective in mitigating the harmful influence of salinity-induced osmotic and oxidative stress than their individual application.

## Conclusion

As compared to individual applications of the studied organic amendments (compost, farmyard manure and press mud), their different combination (i.e. 2.5% (FYM + press mud), 5% press mud, and 10% ) have higher potential to alleviate adverse effects of salinity-induced osmotic and oxidative stresses in okra plants thus combining these organic fertilizers can be a more practical approach to improve salinity-induced osmotic and oxidative stress tolerance in okra, which will lead to improved crop yield under saline condition.

## Data Availability

Data will be available on personal request from corresponding author.

## Abbreviations

*A*: Absorbance
CAT: Catalase
E: Transpiration rate
EDTA: Ethylenediaminetetraacetic acid
FYM: Farmyard manure
g: Gram
H_2_O_2_: Hydrogen peroxide
IRGA: Infra–red gas analyzer
IU: Enzyme activity
K: Kelvin
Kg: Kilogram
MA: Molar absorption
µL: Microliter
µmol: Micromole
mbars: Millibars
MDA: Malondialdehyde
mL: Milliliter
mm: Millimetre
mM: Millimolar
Mol: Mole
MPa: Megapascal
nm: Nanometer
NPK: Nitrogen, phosphorus and potassium
PAR: Photosynthetically active radiation
PCA: Principal component analysis
PM: Press mud
P_net_: Net photosynthetic rate
POD: Peroxidase
ROS: Reactive oxygen species
SOD: Superoxide dismutase
T: Temperature
TBARS: Thiobarbituric acid reactive substances
UV: Vis–ultra violet–visible
v/v: Volume per volume
WUE: Vater use efficiency
w/v: Veight per volume
IU: International unit of enzyme activity

## References

[1] Sultan I, Khan I, Chattha MU, Hassan MU, Barbanti L, Calone R, Izzat W (2021). Improved salinity tolerance in early growth stage of maize through salicylic acid foliar application. Ital J Agron.

[2] Abdel Latef AAH, Abu Alhmad MF, Kordrostami M, Abo-Baker ABAE, Zakir A (2020). Inoculation with *Azospirillum lipoferum* or *Azotobacter chroococcum* reinforces maize growth by improving physiological activities under saline conditions. J Plant Growth Regul.

[3] Kamran M, Parveen A, Ahmar S, Malik Z, Hussain S, Chattha MS, Saleem MH, Adil M, Heidari P, Chen JT (2020). An overview of hazardous impacts of soil salinity in crops, tolerance mechanisms and amelioration through selenium supplementation- A review. Int J Mol Sci.

[4] Mustafa A, Saeed Q, Nezhad MTK, Nan S, Hongjun G, Ping Z, Minggang X (2023). Physically separated soil organic matter pools as indicators of carbon and nitrogen change under long-term fertilization in a Chinese Mollisol. Environ Res.

[5] Qari SH, Hassan MU, Chattha MU, Mahmood A, Naqve M, Nawaz M, Barbanti L, Alahdal MA, Aljabri M (2022). Melatonin induced cold tolerance in plants: physiological and molecular responses. Front Plant Sci.

[6] Singhal RK, Saha D, Skalicky M, Mishra UN, Chauhan J, Behera LP (2021). Crucial cell signaling compounds crosstalk and integrative multi-omics techniques for salinity stress tolerance in plants. Front Plant Sci.

[7] Mustafa A, Hu X, Abrar MM, Shah SAA, Nan S, Saeed Q, Minggang X (2021). Long-term fertilization enhanced carbon mineralization and maize biomass through physical protection of organic carbon in fractions under continuous maize cropping. Appl Soil Ecol.

[8] Fileccia V, Ruisi P, Ingraffia R, Giambalvo D, Frenda AS, Martinelli F (2017). Arbuscular mycorrhizal symbiosis mitigates the negative effects of salinity on durum wheat. PLoS ONE.

[9] Hassan MU, Chattha MU, Khan I, Chattha MB, Barbanti L, Aamer M (2021). Heat stress in cultivated plants: Nature, impact, mechanisms, and mitigation strategies—A review. Plant Biosys.

[10] Batool M, El-Badri AM, Wang Z, Mohamed IA, Yang H, Ai X, Wang B (2022). Rapeseed morpho-physio-biochemical responses to drought stress induced by PEG-6000. Agronomy.

[11] Oustric J, Morillon R, Ollitrault P, Herbette S, Luro F, Froelicher Y, Tur I, Dambier D, Giannettini J, Berti L, Santini J (2018). Somatic hybridization between diploid Poncirus and Citrus improves natural chilling and light stress tolerances compared with equivalent doubled-diploid genotypes. Trees – Struct Funct.

[12] Siddiqui MN, Mostofa MG, Akter MM, Srivastava AK, Sayed MA, Hasan MS, Tran L-SP (2017). Impact of salt-induced toxicity on growth and yield-potential of local wheat cultivars: oxidative stress and ion toxicity are among the major determinants of salt-tolerant capacity. Chemosphere.

[13] Mustafa A, Holatko J, Hammerschmiedt T, Kucerik J, Kintl A, Baltazar T, Brtnicky M. The role of biochar co-pyrolyzed with sawdust and zeolite on soil microbiological and physicochemical attributes, crop agronomic, and ecophysiological performance. J Soil Sci Plant Nut. 2023;23:1–13. 10.1007/s42729-023-01428-8.

[14] Zafar SA, Patil SB, Uzair M, Fang J, Zhao J, Guo T, Yuan S, Uzair M, Luo Q, Shi J, Schreiber L, Li X (2019). Degenerated panicle and partial sterility 1 (DPS1) encodes a CBS domain containing protein required for anther cuticle and panicle development in rice. New Phytol.

[15] Mahmood U, Hussain S, Hussain S, Ali B, Ashraf U, Zamir S, Al-Robai SA, Alzahrani FO, Hano C, El-Esawi MA (2021). Morpho-Physio-biochemical and molecular responses of maize hybrids to salinity and waterlogging during stress and recovery phase. Plants.

[16] Evelin H, Devi TS, Gupta S, Kapoor R (2019). Mitigation of salinity stress in plants by arbuscular mycorrhizal symbiosis: current understanding and new challenges. Front Plant Sci.

[17] Annunziata MG, Ciarmiello LF, Woodrow P, Maximova E, Fuggi A, Carillo P (2017). Durum wheat roots adapt to salinity remodeling the cellular content of nitrogen metabolites and sucrose. Front Plant Sci.

[18] Sami F, Yusuf M, Faizan M, Faraz A, Hayat S (2016). Role of sugars under abiotic stress. Plant Physiol Biochem.

[19] Wu X, Peng J, Liu P, Bei Q, Rensing C, Li Y, Yuan H, Liesack W, Zhang F, Cui Z (2021). Metagenomic insights into nitrogen and phosphorus cycling at the soil aggregate scale driven by organic material amendments. Sci Total Environ.

[20] Chávez-García E, Siebe C (2019). Rehabilitation of a highly saline-sodic soil using a rubble barrier and organic amendments. Soil Tillage Res.

[21] Hassan M, Aamer M, Chattha MU, Haiying T, Khan I, Seleiman MF, Aslam MT (2021). Sugarcane distillery spent wash (dsw) as a bio-nutrient supplement: a win-win option for sustainable crop production. Agronomy.

[22] Amin OAHE, El-kersh MAM, Azooz MM (2020). Application of hemin-induced growth and biochemical modifications in Hassawi okra (*Abelmoschus esculentus* L.) grown in seawater salinity. Aust J Crop Sci.

[23] Alabdallah NM, Alzahrani HS (2020). The potential mitigation effect of ZnO nanoparticles on (*Abelmoschus esculentus* L. Moech) metabolism under salt stress conditions. Saudi J Biol Sci.

[24] Capell B, Doerffling K (1993). Genotype specific differences in chilling tolerance of maize in relation to chilling induced changes in water status and abscisic acid accumulation. Physiol Plant.

[25] DuBois M, Gilles KA, Hamilton JK, Rebers PA, Smith F (1956). Colorimetric method for determination of sugars and related substances. Anal Chem.

[26] Bates LS, Waldren RP, Teare ID (1973). Rapid determination of free proline for water stress studies. Plant Soil.

[27] Lowry OH, Rosebrough NJ, Farr AL, Randall RJ (1951). Protein measurement with the Folin phenol reagent. J Biol Chem.

[28] Prochazkova D, Sairam RK, Srivastava GC, Singh DV (2001). Oxidative stress and antioxidant activity as the basis of senescence in maize leaves. Plant Sci.

[29] Du Z, Bramlage WJ (1992). Modified thiobarbituric acid assay for measuring lipid oxidation in sugar-rich plant tissue extracts. J Agric Food Chem.

[30] Beauchamp C, Fridovich I (1971). Superoxide dismutase. Improved assays and an assay applicable to acrylamide gel. Anal Biochem.

[31] Teranishi Y, Tanaka A, Osumi M, Fukui S (1974). Catalase activity of hydrocarbon utilizing Candida yeast. Agric Biol Chem.

[32] Patti F, Bonet-Maury P (1953). Méthode colorimétrique pour le dosage de la catalase. Bull Soc Chem Biol.

[33] Vetter JL, Steinberg MP, Nelson AL (1958). Quantitative determination of peroxidase in sweet corn. J Agric Food Chem.

[34] Gorin N, Heidema FT (1976). Peroxidase activity in golden delicious apples as a possible parameter of ripening and senescence. J Agric Food Chem.

[35] Analytical software (2005). Statistics 8.1 ' ‘User’s Manual.

[36] Ansari M, Shekari F, Mohammadi MH, Juhos K, Végvári G, Biró B (2019). Salt-tolerant plant growth-promoting bacteria enhanced salinity tolerance of salt-tolerant alfalfa (*Medicago sativa* L.) cultivars at high salinity. Acta Physiol Plant.

[37] Orozco-Mosqueda MDC, Glick BR, Santoyo G (2020). ACC deaminase in plant growth-promoting bacteria (PGPB): an efficient mechanism to counter salt stress in crops. Microbiol Res.

[38] Javed Q, Wu Y, Xing D, Ullah I, Azeem A, Rasool G (2018). Salt-induced effects on growth and photosynthetic traits of *Orychophragmus violaceus* and its restoration through re-watering. Braz J Bot.

[39] Azeem A, Wu Y, Javed Q, Xing D, Ikram Ullah I, Kumi F (2017). Response of okra based on electrophysiological modeling under salt stress and re-watering. Biosci J (Uberlândia).

[40] Wani AS, Ahmad A, Hayat S, Fariduddin Q (2013). Salt-induced modulation in growth, photosynthesis and antioxidant system in two varieties of *Brassica juncea*. Saudi J Biol Sci.

[41] Wang S, Liu P, Chen D, Yin L, Li H, Deng X (2015). Silicon enhanced salt tolerance by improving the root water uptake and decreasing the ion toxicity in cucumber. Front Plant Sci.

[42] Sadak MS, Abdelhamid MT (2015). Influence of amino acids mixture application on some biochemical aspects, antioxidant enzymes and endogenous polyamines of *Vicia faba* plant grown under seawater salinity stress. Gesunde Pflanz.

[43] Tartoura KAH, Youssef SA, Tartoura ESAA (2014). Compost alleviates the negative effects of salinity via up-regulation of antioxidants in *Solanum lycopersicum* L. plants. Plant Growth Regul.

[44] Babitha KC, Vemanna RS, Nataraja KN, Udayakumar M (2015). Overexpression of EcbHLH57 transcription factor from *Eleusine coracana* L. in Taco confer tolerance to salt, oxidative and drought stress. PLoS ONE.

[45] Hasanuzzaman M, Bhuyan MHM, Nahar K, Hossain MD, Mahmud JA, Hossen M (2018). Potassium: a vital regulator of plant responses and tolerance to abiotic stresses. Agronomy.

[46] Dustgeer Z, Seleiman MF, Khan I, Chattha MU, Alhammad BA, Jalal RS (2021). Glycine-betaine induced salinity tolerance in maize by regulating the physiological attributes, antioxidant defense system and ionic homeostasis. Not Bot Horti Agrobot Cluj-Napoca.

[47] Fatima A, Hussain S, Hussain S, Ali B, Ashraf U, Zulfiqar U (2021). Differential morpho-physiological, biochemical, and molecular responses of maize hybrids to salinity and alkalinity stresses. Agronomy.

[48] Naveed M, Ramzan N, Mustafa A, Samad A, Niamat B, Yaseen M, Ahmad Z, Hasanuzzaman M, Sun N, Shi W, Xu M (2020). Alleviation of salinity induced oxidative stress in *Chenopodium quinoa* by Fe biofortification and biochar–endophyte interaction. Agronomy.

[49] Al-Taey DK, Majid ZZ (2018). Study effect of kinetin, bio-fertilizers and organic matter application in lettuce under salt stress. J Glob Pharma Technol.

[50] Kusvuran A, Bilgici M, Kusvuran S, Nazli RI (2021). The effect of different organic matters on plant growth regulation and nutritional components under salt stress in sweet sorghum (*Sorghum bicolor* (L.) Moench). Maydica.

[51] Bziouech SA, Dhen N, Helaoui S, Ammar IB, Dridi BAM (2022). Effect of vermicompost soil additive on growth performance, physiological and biochemical responses of tomato plants (*Solanum lycopersicum* L. var. Firenze) to salt stress. Emir J Food Agric.

[52] Alamer KH, Perveen S, Khaliq A, Zia Ul Haq M, Ibrahim MU, Ijaz B (2022). Mitigation of salinity stress in maize seedlings by the application of vermicompost and sorghum water extracts. Plants.

[53] Sheoran P, Kumar A, Singh A, Kumar A, Parjapat K, Sharma R (2021). Pressmud alleviates soil sodicity stress in a rice–wheat rotation: effects on soil properties, physiological adaptation and yield-related traits. Land Degrad Dev.

[54] Islam F, Yasmeen T, Arif MS, Ali S, Ali B, Hameed S, Zhou W (2016). Plant growth promoting bacteria confer salt tolerance in *Vigna radiata* by up-regulating antioxidant defense and biological soil fertility. Plant Growth Regul.

[55] Hozayn M, Abdel-Monem AA, Ebtihal MAEM, Abdul Qados AMS (2013). Amelioration of salinity stress in mung bean (*Vigna radiata* L.) plant by soaking in arginine. J Appl Sci Res.

[56] Kausar F, Shahbaz M, Ashraf M (2013). Protective role of foliar-applied nitric oxide in *Triticum aestivum* under saline stress. Turk J Bot.

[57] Revathi S, Pillai MA (2015). Novel approach in screening rice genotype for tolerance to salt stress under hydroponic culture. Agric Sci Digest.

[58] Amiri A, Baninasab B, Ghobadi C, Khoshgoftarmanesh AH (2016). Zinc soil application enhance photosynthetic capacity and antioxidant enzyme activities in almond seedlings affected by salinity stress. Photosynthetica.

[59] Yaghoubian I, Antar M, Ghassemi S, Modarres-Sanavy SAM, Smith DL (2022). The Effects of Hydro-Priming and Colonization with Piriformospora indica and Azotobacter chroococcum on Physio-Biochemical Traits, Flavonolignans and Fatty Acids Composition of Milk Thistle (Silybum marianum) under Saline Conditions. Plants.

[60] Hossain MA, Mostofa MG, Fujita M (2013). Cross protection by cold-shock to salinity and drought stress-induced oxidative stress in mustard (*Brassica campestris* L.) seedlings. Mol Plant Breed.

[61] Gupta B, Huang B (2014). Mechanism of salinity tolerance in plants: physiological, biochemical, and molecular characterization. Int J Genom.

[62] Singh M, Kumar J, Singh S, Singh VP, Prasad SM (2015). Roles of osmoprotectants in improving salinity and drought tolerance in plants: a review. Rev Environ Sci Bio.

[63] Azooz MM, Metwally A, Abou-Elhamd MF (2015). Jasmonate-induced tolerance of hassawi okra seedlings to salinity in brackish water. Acta Physiol Plant.

[64] Isayenkov SV, Maathuis FJ (2019). Plant salinity stress: many unanswered questions remain. Front Plant Sci.

[65] Farouk S, Al-Huqail AA (2020). Sodium nitroprusside application regulates antioxidant capacity, improves phytopharmaceutical production and essential oil yield of marjoram herb under drought. Indust Crops Prod.

